# Potential Dependent Reorientation Controlling Activity of a Molecular Electrocatalyst

**DOI:** 10.1021/jacs.3c13076

**Published:** 2024-03-05

**Authors:** Adrian M. Gardner, Gaia Neri, Bhavin Siritanaratkul, Hansaem Jang, Khezar H. Saeed, Paul M. Donaldson, Alexander J. Cowan

**Affiliations:** † Department of Chemistry and Stephenson Institute for Renewable Energy, University of Liverpool, Liverpool L69 7ZD, United Kingdom; ‡ Early Career Laser Laboratory, University of Liverpool, Liverpool L69 3BX, United Kingdom; § Central Laser Facility, Research Complex at Harwell, STFC Rutherford Appleton Laboratory, Didcot, Oxfordshire OX11 0QX, United Kingdom

## Abstract

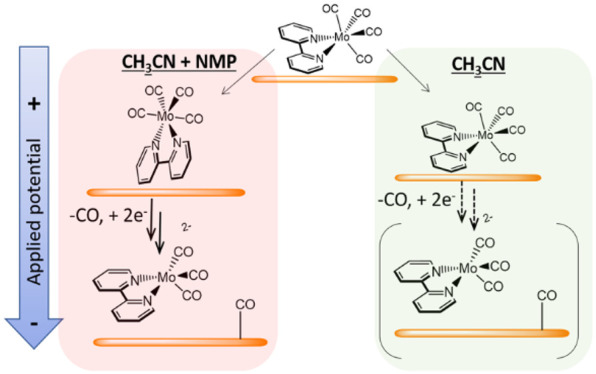

The activity of molecular electrocatalysts depends on the interplay of electrolyte composition near the electrode surface, the composition and morphology of the electrode surface, and the electric field at the electrode–electrolyte interface. This interplay is challenging to study and often overlooked when assessing molecular catalyst activity. Here, we use surface specific vibrational sum frequency generation (VSFG) spectroscopy to study the solvent and potential dependent activation of Mo(bpy)(CO)_4_, a CO_2_ reduction catalyst, at a polycrystalline Au electrode. We find that the parent complex undergoes potential dependent reorientation at the electrode surface when a small amount of *N*-methyl-2-pyrrolidone (NMP) is present. This preactivates the complex, resulting in greater yields at less negative potentials, of the active electrocatalyst for CO_2_ reduction.

There is interest in developing new transition metal electrocatalysts for a range of applications related to devices for fuel generation/consumption and energy storage. An advantage of molecular electrocatalysts is that they are synthetically tunable, and in contrast to the more complex enzymatic centers from which inspiration is often drawn, their simplicity in principle allows for the evaluation of molecular structure-function relationships.^1−3^ In addition to the role of the catalyst’s structure on activity, important but often overlooked is how the molecular catalyst interacts with the electrode surface and the impact of the potential dependent composition of the electrolyte (solvent, supporting electrolyte) near the electrode surface. Interfacial electric field effects and the subsequent impact of double layer structure on heterogeneous catalysts/metal electrodes are widely discussed to rationalize the behavior of cation induced activity changes, for example, during CO_2_ reduction.^4−6^ However, direct measurement of potential/field and solvent induced rearrangements of molecular electrocatalysts is rarer, despite it being reasonable to assume that there is impact on activity.^7−9^

Past studies by Hartl and colleagues have shown that the activity of the CO_2_ reduction electrocatalyst Mo(bpy)(CO)_4_ has a strong dependence on the nature of the electrode material and solvent.^2,10^ The active catalytic species that binds CO_2_ is Mo(bpy)(CO)_3_^2–^ (Figure 1a).^10^ Using vibrational sum frequency generation (VSFG) spectroscopy, we have previously shown that when CH_3_CN is the solvent a small population of this species is formed specifically on Au surfaces at potentials positive of those anticipated from the diffusion controlled redox potential,^11^ explaining the improved catalytic activity at Au. The solvent dependent behavior of this catalyst is not yet understood. Specifically, NMP (*N*-methyl-2-pyrrolidone) is known to enhance catalytic activity.^10^

**Figure 1:**
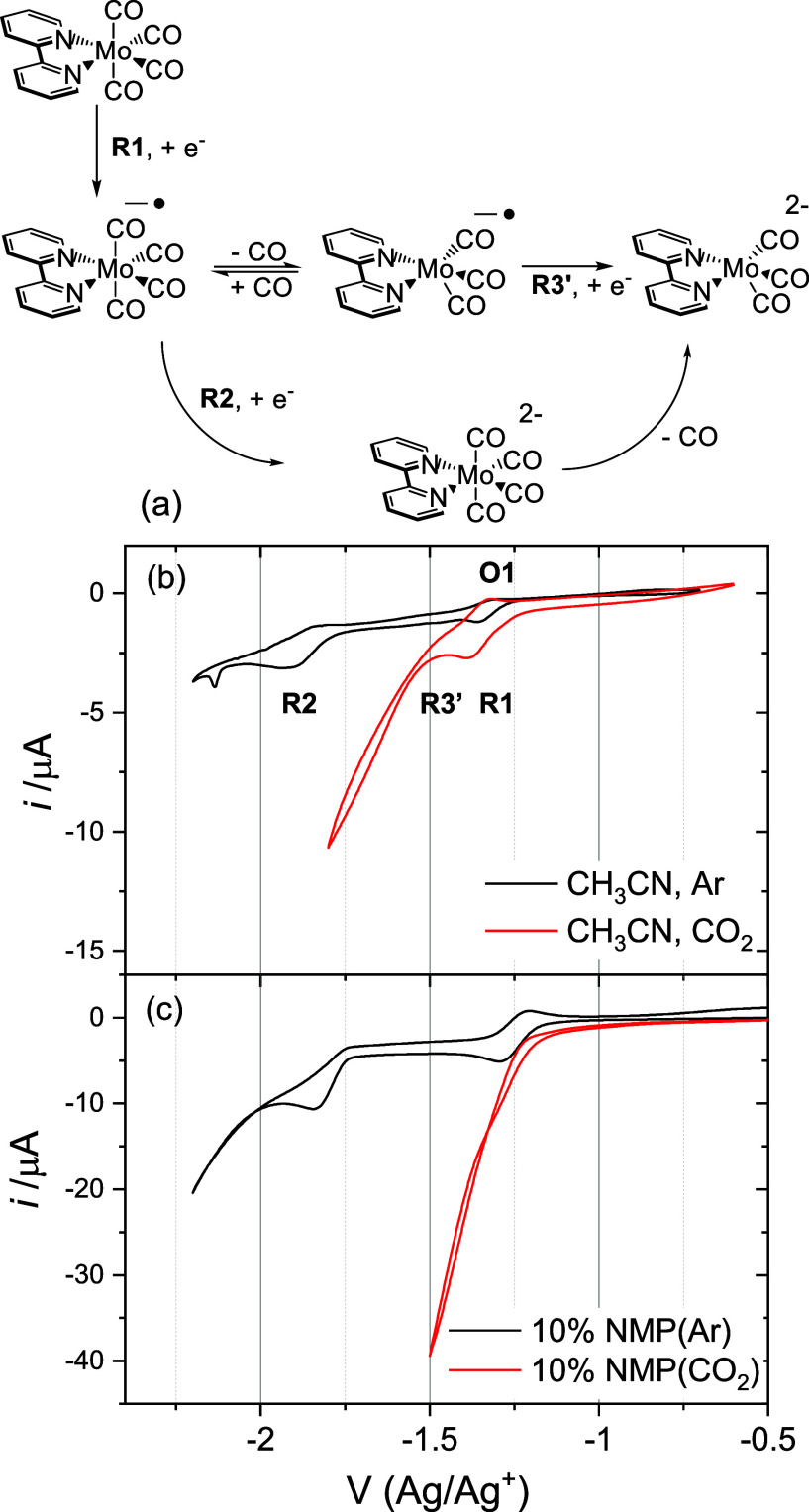
(a) Mechanism of formation of the active catalyst Mo(bpy)(CO)_3_^2–^ in CH_3_CN where a small population of Mo(bpy)(CO)_4_^–^ undergoes CO loss on Au and facile reduction (R3′) to form Mo(bpy)(CO)_3_^2–^. CVs (20 mV s^–1^) of Mo(bpy)(CO)_4_ (1 mM) in CH_3_CN (b) and CH_3_CN with 10% (vol.) NMP (c), both with 0.1 M TBAPF_6_ at Au electrode under Ar (black) and CO_2_ (red).

Cyclic voltammograms (CVs) of Mo(bpy)(CO)_4_ at a Au electrode in CH_3_CN and CH_3_CN with 10% (vol.) NMP are shown in Figure 1b,c. Mo(bpy)(CO)_4_ gives rise to a catalytic current for CO_2_ reduction in both solvents; however, the onset is at more positive potentials, and the magnitude of the catalytic current is greater when 10% NMP is added to the solution. Neat NMP has previously been shown to enhance the CO_2_ reduction current.^10^ The observation that even 10% NMP in CH_3_CN can replicate this enhancement means it is not a result of the bulk solvent properties of NMP and is instead proposed to be due to the way that NMP can interact with the electrocatalyst or the electrode surface.

Figure 2 shows VSFG spectra for the same polycrystalline Au electrode in a solution of Mo(bpy)(CO)_4_ in CH_3_CN and CH_3_CN with 10% NMP under Ar as the potential is swept from approximately the open circuit voltage to negative of reduction 1 “R1”; see Supporting Information Note 1 for experimental details. The assignment of the VSFG spectra of Mo(bpy)(CO)_4_ in CH_3_CN has been reported previously^11^ and is briefly covered here; see Supporting Information Note 2 for full details. Initially at −0.55 V, the spectrum is dominated by a band at ∼1890 cm^–1^ due to a ν(CO) mode of Mo(bpy)(CO)_4_, with the complex accumulating as the electrode is made more negative, Figure 2a.^10,11^ Between −0.65 and −1.15 V, the 1890 cm^–1^ ν(CO) band of Mo(bpy)(CO)_4_ shifts in frequency with applied potential (4.5 ± 0.7 cm^–1^ V^–1^, Figure 3) demonstrating that the complex is at or near the electrode surface (within the double layer structure).^12,13^ Weaker bands at 1905 and ∼1830 cm^–1^ and a shoulder at ∼1870 cm^–1^ are also present between −0.55 and −1.15 V, Figure S3. The band at 1905 cm^–1^ is due to a concentration of Au–CO (Figure S3) while the weak bands at ∼1830 and 1870 cm^–1^ are assigned to Mo(bpy)(CO)_4_.^11^

**Figure 2:**
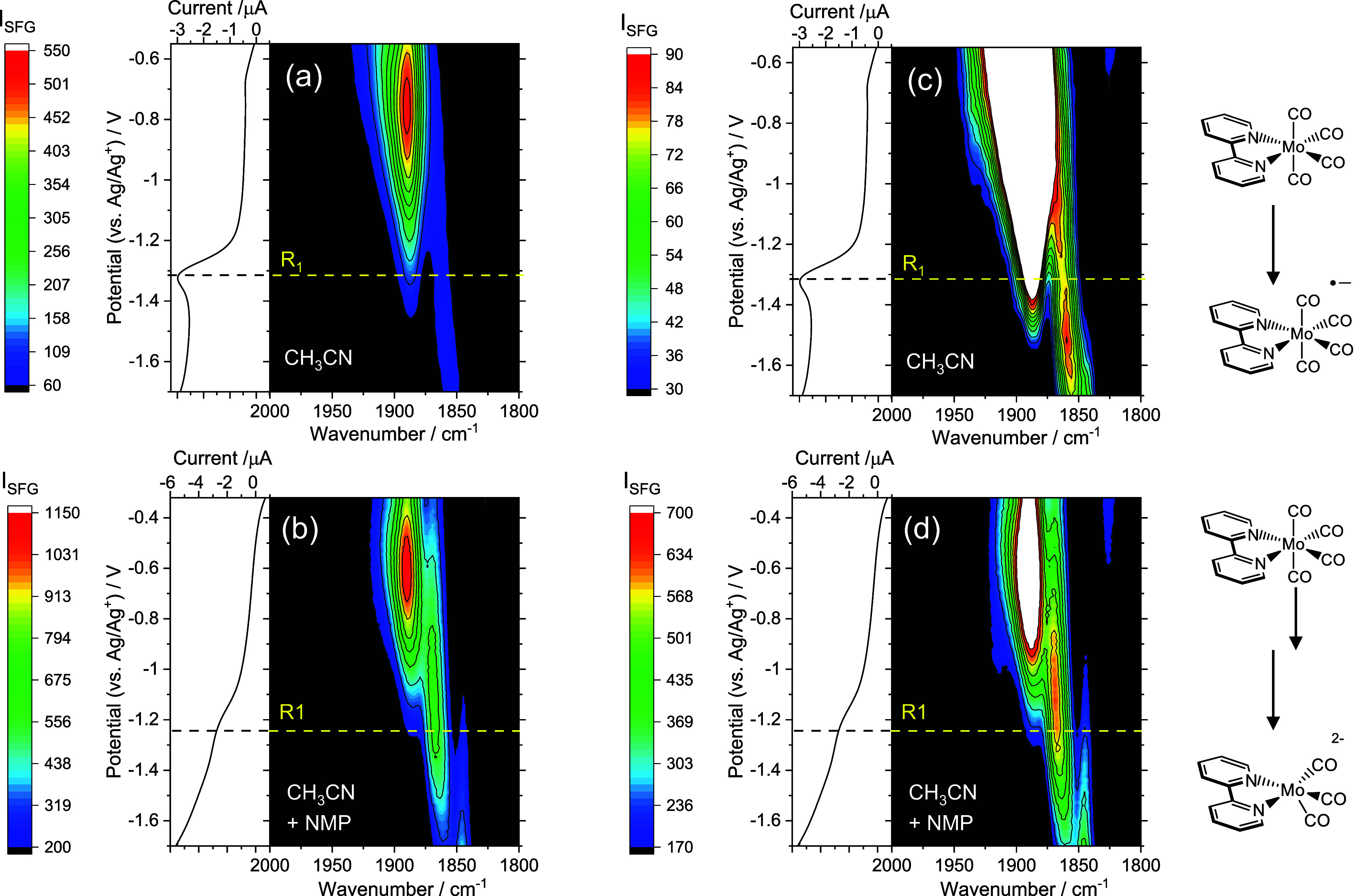
VSFG spectra in the ν(CO) region of Mo(bpy)(CO)_4_ (1 mM) in CH_3_CN (a, c) and CH_3_CN with 10% (vol.) NMP (b, d) during a linear sweep (5 mV s^–1^, CH_3_CN (a, c) and 2.5 mV s^–1^ 10% (vol.) NMP (b, d) positive to negative direction) with 0.1 M TBAPF_6_ at a Au electrode under Ar, *ppp* polarization. Parts c and d are replotted over a limited SFG intensity range. The chemical structures on the right-hand side represent the dominant species to which the VSFG spectra are assigned.

**Figure 3:**
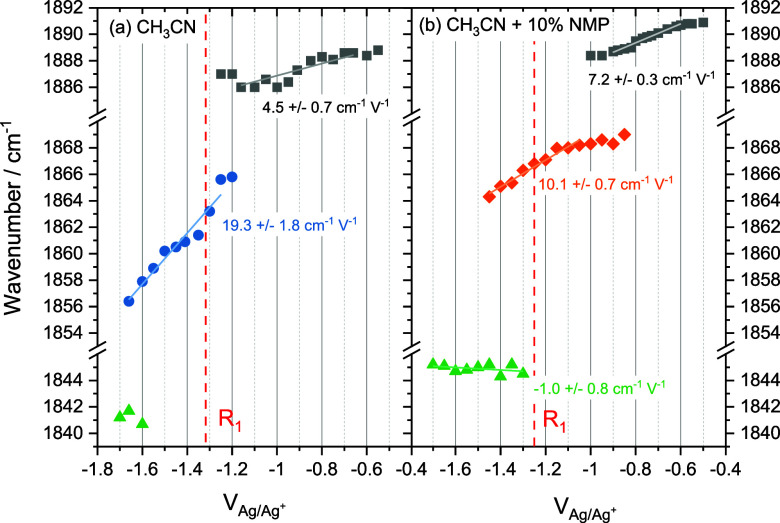
Potential dependent ν(CO) frequencies during VSFG experiments of Mo(bpy)(CO)_4_ in CH_3_CN (a) and with 10% NMP (b). Gray squares, Mo(bpy)(CO)_4_; blue circles, Mo(bpy)(CO)_4_^–^; green triangles, Mo(bpy)(CO)_3_^2–^. We demonstrate below that the orange diamonds in (b) are due to reoriented Mo(bpy)(CO)_4_. The span of the fit lines indicates the potential range used to generate the tuning rate.

At approximately −1.3 V, reduction of Mo(bpy)(CO)_4_ occurs (R1, Figures 1 and 2). The band at ∼1890 cm^–1^ decreases, and a new band at 1863 cm^–1^ grows (tuning rate 19.3 ± 1.8 cm^–1^ V^–1^, Figure 3), assigned to the reduced species Mo(bpy)(CO)_4_^–^.^10,11^ At −1.5 V, when the VSFG spectra are replotted over a limited *I*_VSFG_ range (Figure 2c), a weak band assignable to Mo(bpy)(CO)_3_^2–^ is detected (∼1841 cm^–1^). This is due to a small population of Mo(bpy)(CO)_4_^–^ undergoing CO loss and subsequent reduction at the Au electrode surface.^11^

When NMP is present, positive of −0.6 V, the VSFG spectra (Figure 2b) are similar to those recorded in CH_3_CN alone, with a dominant band at ∼1890 cm^–1^ and weaker bands at ca. 1830 and 1870 cm^–1^, assignable to ν(CO) modes of Mo(bpy)(CO)_4_. We conclude that the orientation and nature of interaction between Mo(bpy)(CO)_4_ and the Au surface positive of −0.6 V are similar in both the presence and absence of NMP.

Negative of −0.6 V, the VSFG spectra in the presence of NMP are markedly different. The ∼1890 cm^–1^ Mo(bpy)(CO)_4_ VSFG band decreases, and a band at ∼1870 cm^–1^ (−0.85 V, 10.1 ± 0.7 cm^–1^ V^–1^) grows, becoming dominant by −1.0 V. The decrease in intensity at ∼1890 cm^–1^ does not correlate with a measured change in current *in situ*. Separate CV and square wave voltammetry (SWV, Figure 4) measurements also do not show any clear features in the potential window of interest (−0.6 to −1.0 V) assignable to Faradaic processes. We performed differential capacitance measurements of CH_3_CN and CH_3_CN + 10% NMP, as shown in Figure 4c. These use a low concentration of TBAPF_6_ (0.1 mM) to enable observation of the point of zero charge (pzc) and potentials where specific ion/solvent absorption occurs^14^ in the absence of Mo(bpy)(CO)_4_. The potential at pzc is approximately ∼0.6 V for both solvent compositions, but the capacitance is different at potentials positive of pzc. With NMP, we observe a broadening and shift to −0.6 V in the capacitance peak, coinciding with the potential where there is an abrupt change in spectral activity (Figure 2). This indicates a potential dependent change in the double layer structure owing to the presence of a small amount (10% vol.) of NMP.

**Figure 4:**
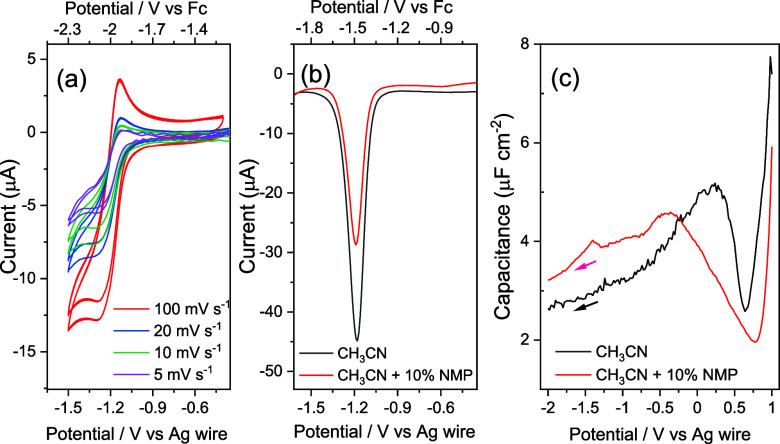
(a) Variable scan rate CV of R1 of Mo(bpy)(CO)_4_ in CH_3_CN with 10% NMP (b) SWV of R1 at 100 mV s^–1^. Both (a) and (b) use 100 mM TBAPF_6_. (c) Differential capacitance of the Au electrode in the presence and absence of NMP with 0.1 mM TBAPF_6_ recorded at 10 Hz +1 to −2 V vs Ag.

With no Faradaic processes to explain the loss in VSFG intensity when NMP is present, we conclude that the change in the VSFG spectra is due to reorientation of Mo(bpy)(CO)_4_ at the electrode surface between −0.6 and −1.0 V as a result of the potential and solvent dependent double layer restructuring. The intensity of VSFG modes is dependent upon their relative orientation, and we assign a reorientation of Mo(bpy)(CO)_4_ as the cause of the decrease in the relative intensity of the ∼1890 cm^–1^ band and increase in intensity of the 1870 cm^–1^ band. Supporting this assignment is the observation that the 1870 cm^–1^ band persists until R1 (−1.25 V, Figure 2b) and that during CVs (−0.3 to −1.7 to −0.3 V, Figure S2) the 1870 cm^–1^ band reforms at potentials slightly positive of O1 (−1.2 V, Figure 1a). Reorientation of interfacial species as a result of changing field^15^ and non-Faradaic potential dependent reactions^16^ has been previously reported. Owing to the agreement in potential of R_1_ in the presence and absence of NMP (Figure 1) and the abrupt change in spectral activity in the vicinity R_1_ (Figure 2), we exclude the possibility that we observe a non-Faradaic transient product prior to R_1_ in the presence of NMP in this work.

In the presence of NMP at potentials negative of R1 (−1.25 V), a new band at 1845 cm^–1^ grows as the band at 1870 cm^–1^ of Mo(bpy)(CO)_4_ decays, Figure 2. Through correlation of its appearance at potentials where catalysis onsets in NMP (Figure 1) and through spectroelectrochemical studies,^10,11^ we assign the band at 1845 cm^–1^ to Mo(bpy)(CO)_3_^2–^. By −1.6 V, Mo(bpy)(CO)_3_^2–^, the active CO_2_ reduction catalyst, is the dominant species at the electrode when NMP is present. In contrast, in CH_3_CN at −1.6 V, Mo(bpy)(CO)_4_^–^ is dominant and the catalytic current is significantly lower.

We propose that, in the presence of NMP, reorientation of Mo(bpy)(CO)_4_ facilitates rapid (likely subsecond) CO loss from the initially formed Mo(bpy)(CO)_4_^–^, leading to Mo(bpy)(CO)_3_^–^, which is immediately reduced to Mo(bpy)(CO)_3_^2–^, consistent with the irreversibility of the R_1_/O_1_ redox couple in CVs at <100 mV s^–1^, Figure 4a. The reduction potential of Mo(bpy)(CO)_3_^–^ has not been determined experimentally, but DFT calculations, albeit without explicit solvent or the presence of the electrode and local electric field, show that Mo(bpy)(CO)_3_^–^ reduction occurs at potentials positive of Mo(bpy)(CO)_4_^–^,^17^ supporting the proposed mechanism. It is known that photochemical ligand substitution of this class of complexes occurs at the axial sites,^18^ and it can be hypothesized that CO loss is facilitated from the tetracarbonyl by reorientation to make the axial CO groups accessible by making them parallel to the Au surface to enable interaction between the carbon atom and the electrode. In CH_3_CN alone, we observe only a slight change in the relative *I*_VSFG_ of the Mo(bpy)(CO)_4_ bands as the potential is changed (Figure S4), indicating that the applied potential is having less effect on the complex (see Note S2) and the lack of reorientation hinders CO loss from Mo(bpy)(CO)_4_^–^.

In conclusion, the large increase in electrocatalytic activity of Mo(bpy)(CO)_4_ when a small (10% vol.) amount of NMP is added to an electrolyte solution is shown to be due to a potential dependent solvent restructuring of the double layer that leads to reorientation of Mo(bpy)(CO)_4_ at the electrode surface. This preactivates the complex and leads to a greater yield at less negative potentials of the active catalyst for CO_2_ reduction (Mo(bpy)(CO)_3_^2–^). Determining the actual orientation of the complex by simulation is beyond the state-of-the-art computational methodologies. This would require development of highly complex models of the double layer under potentiostatic control that includes explicit solvent, electrolyte ions, and catalyst interactions on the complicated polycrystalline charged Au surface. The experiments reported here and, previously,^11^ show that the geometry and structure of the molecular catalyst is dependent on all of these interactions, highlighting the complex nature of such studies. Until such *in silico* models can be realized, *in situ* spectroscopic studies that specifically target the electrode interface remain vital for rationalizing electrocatalytic activity.

## Supporting Information Available

Experimental details; detailed discussion of spectral assignments (PDF)
