# Supplementary material for: Potential Dependent Reorientation Controlling Activity of a Molecular Electrocatalyst
Source: J Am Chem Soc. 2024 Mar 5;146(11):7130–4. doi: 10.1021/jacs.3c13076 (PMC10958496; doi:10.1021/jacs.3c13076)
Supplement: Supplementary file 1 — ja3c13076_si_001.pdf [file ja3c13076_si_001.pdf]

Electronic Supplementary Information for:

Potential dependent reorientation controlling activity of a molecular electrocatalyst

Adrian M. Gardner,[a,b] Gaia Neri,[a†] Bhavin Siritanaratkul,[a] Hansaem Jang,[a] Khezar H. Saeed,[a‡] Paul M. Donaldson[c] and Alexander J. Cowan[a*]

Addresses

[a] Department of Chemistry and Stephenson Institute for Renewable Energy, University of Liverpool, Liverpool, L69 7ZD, United Kingdom. acowan@liverpool.ac.uk

[b] Early Career Laser Laboratory, University of Liverpool, Liverpool, L69 3BX , United Kingdom.

[c] Central Laser Facility, Research Complex at Harwell, STFC Rutherford Appleton Laboratory, Didcot, Oxfordshire OX11 0QX, United Kingdom.

[†] Current address - Johnson Matthey, Sonning Common, Reading, RG4 9NH, United Kingdom.

[‡] Current address -Department of Chemistry, Aarhus University, Langelandsgade 140, 8000, Aarhus, Denmark.

Supplementary Note 1: Experimental Details.

In situ Vibrational Sum Frequency Experiments

VSFG is a second order non-linear process that does not occur in the bulk of centrosymmetric media meaning that it is possible to obtain the vibrational spectra of molecular catalysts at, or near the electrode surface,[1,2,3,4,5,6,7] making it an ideal tool to assess the mechanism of solvent and potential dependent catalytic activity.

The VSFG experiments were performed using a newly constructed spectrometer at The University of Liverpool. 90 % of the output PHAROS-PH1-SP (Light Conversion, 1030 nm, 10 kHz, 10 W, 170 fs pulse duration) laser system is used to generate both the nIR and mIR laser pulses required for the VSFG experiments. 1 W of the output is used to generate a time asymmetric narrow-band nIR pulse (1030 nm, 10 Hz, ~1.5 ps, ~13 cm^{-1} linewidth) *via* an etalon (SLS Optics), which is directed to the sample through a half-wave plate (Thorlabs, WPH10M-1030) and polariser (ThorLabs, LPVIS050-MP2) to rotate the light to a horizonal polarisation, and p-polarised w.r.t refection from the sample, at an angle of incidence of ~45°. A 950 nm long pass filter (Thorlabs, FEL0950) is also used to filter out second harmonic 515 nm light generated within the halfwave plate. This nIR pulse is focused by a 30 cm lens (Thorlabs LB1779-B), with the sample placed ~25 cm away from this lens, resulting in an approximate beam diameter at the sample of ~400 µm ($1/e^2$ diameter) with a power of ~15 mW. 8 W of the laser output is used in an IR OPA (Light Conversion, Orpheus-One-HE) to generate the broadband IR beam which

can be tuned across the frequency range of interest (10 kHz, 170 fs pulse duration and ~150 cm^{-1}@1900 cm^{-1}). The mIR output passes through a twisted periscope to switch the polarisation from vertical to the desired horizontal polarisation. The purity of the polarisation is checked using a polariser (Thorlabs, LPMIR050-MP2) which is removed from the beam path prior to VSFG measurement. The mIR beam is focussed onto the sample, with an approximate beam diameter of 250 µm, using a Au parabolic mirror (Thorlabs, MPD249H-M01) at an angle of incidence of ~50° and *p*-polarisation w.r.t reflection from the sample, with a power of ~50 mW. A gas-purge generator largely removes H_2O, CO_2 and other contaminants in the air from the IR beam path. Resulting *p*-polarised VSFG light is directed through 950 (Thorlabs, FES0950) and 900 nm (Thorlabs, FES0900) short pass filters to remove the 1030 nm nIR beam. The beam goes through another polariser (Thorlabs, LPVIS050-MP2) before being focussed through 150 µm slits and into the spectrograph (Andor, Kymera) using a 15 mm focal length lens (Thorlabs, LA1540-B), and is detected on a CCD camera (Andor, iDus416). nIR/mIR delay is introduced using a linear stage (ThorLabs, LTS300C) on the nIR beam path; all spectra reported herein have been obtained with the nIR pulse delayed by ~0.5 ps w.r.t. the mIR pulse to suppress the non-resonant contribution to the spectrum.[8] Each spectrum was accumulated on the CCD for 2 s before reading out. The spectrograph was calibrated using Ne spectral lines and the rovibrational P, Q and R CO_2 branches).

All VSFG experiments were performed in a custom spectroelectrochemical "cross-cell" as used in our previous work.[7] The 1.6 mm diameter Au (IJ Cambria) working electrode was mechanically polished for 10 minutes using 1.0 µm then for a further 10 minutes using 0.05 µm alumina suspension. The electrode was rinsed thoroughly and sonicated in Milli-Q water between each step. The working electrode was then secured into the cross cell with a Ag wire pseudo reference electrode (sanded, rinsed and sonicated before each experiment) and a Pt counter electrode (flame annealed each day). A Teflon spacer (50 µm), was placed between the Au working electrode and window to ensure the a consistent pathlength between measurements. The solution of 1 mM $Mo(bpy)(CO)_4$, 0.1 M $TBAPF_6$, in CH_3CN or CH_3CN with 10% (vol.) NMP and electrochemical cell were purged for 10 minutes with Ar, before solution was transferred under Ar to the spectroelectrochemical cell. Figure S1 shows the FTIR spectra of $Mo(bpy)CO_4$ in the solvents used.

Owing to the intense absorption of NMP at ~1675 cm^{-1} (see Figure S1), the addition of greater volumes of NMP begin to obscure the ν(CO) bands of $Mo(bpy)(CO)_4$, leading to the possibility of phantom transitions in the VSFG spectra to be observed.[9] Owing to the agreement between the catalytic activity in 10 % (vol.) NMP and pure NMP,[10] we conclude the addition of a small volume of NMP replicates the electrochemical behaviour of pure NMP. This indicates that species at the electrochemical interface is unlikely to reflect that found in the bulk, resulting in relatively small concentrations of additives to have profound effects on catalytic activity.

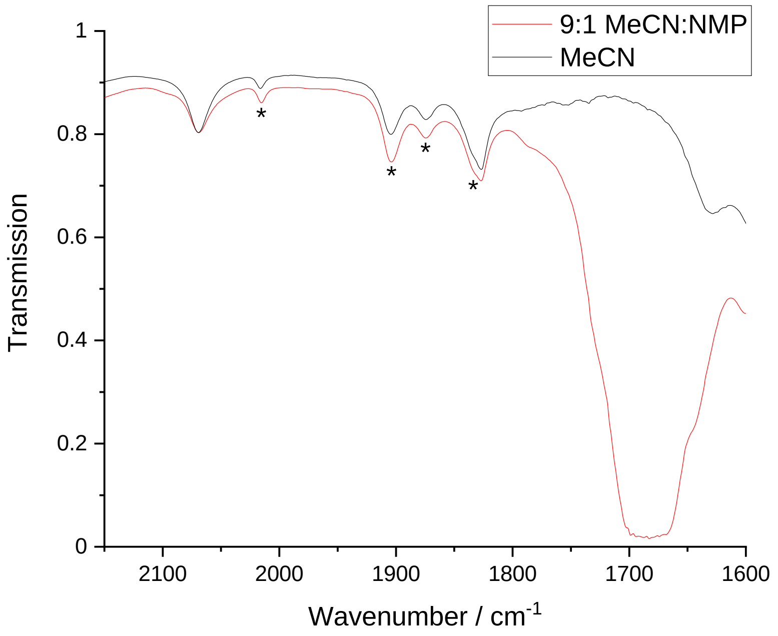

*Figure S1. FTIR spectra of Mo(bpy)(CO)$_4$ (1 mM) in CH_3CN (black) and CH_3CN with 10% (vol.) NMP (red) obtained with a 100 µm pathlength. Bands assignable to ν(CO) modes of Mo(bpy)(CO)$_4$ are marked with *.*

For spectroelectrochemical measurements in pure CH_3CN, the potential was scanned from -0.5 → -2.3 V → -0.5 V at 5 mV s^{-1}. The forward sweep is shown in Figure 2, whereas the full scan range is shown in Figure S1. For CH_3CN with 10% (vol.) NMP, the potential was scanned from -0.3 → -1.5 V → -0.3 V at 2.5 mV s^{-1} again forward sweep is shown in Figure 2, whereas the full scan range is shown in Figure S2. The lack of VSFG bands at potentials more negative than R_2 is likely a result of disorder at the electrode-electrolyte interface. For assignments see supplementary note 2. We have shown that Mo(bpy)CO_4^- undergoes CO loss and is rapidly reduced to Mo(bpy)CO_3^{2-} on a Au electrode,[7] a potentially non-reversible couple.[10] We limit the most negative potential studied to -1.5 V, ~250 mV more negative than the onset of electrocatalysis in the presence of CO_2, and ~300 mV more positive than R_2 (Figure 1).

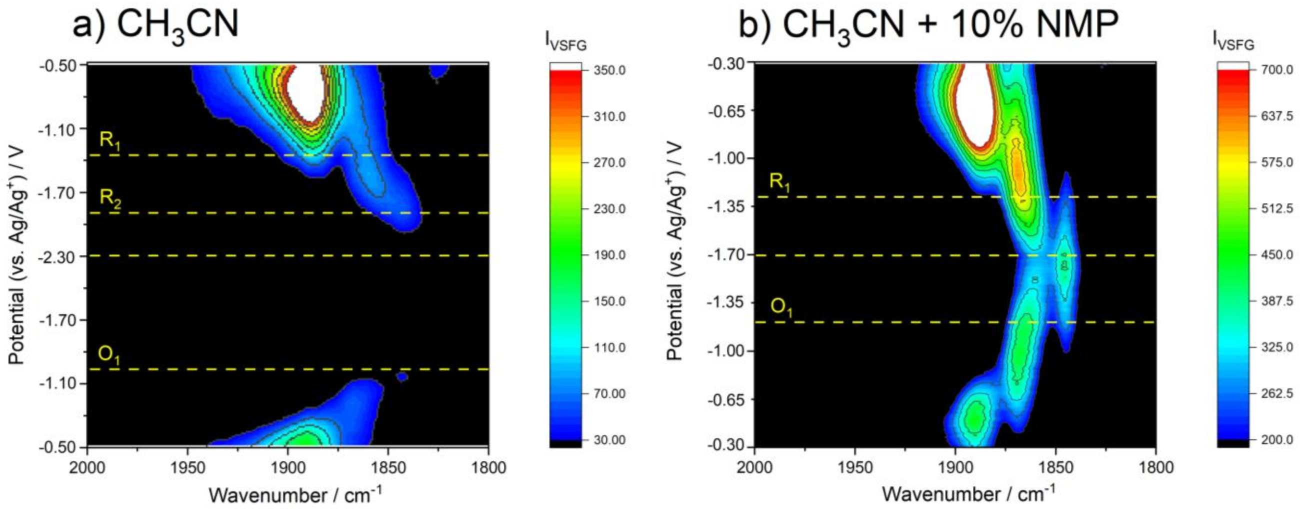

Figure S2. VSFG spectra obtained in the ν(CO)spectral region of Mo(bpy)(CO)$_4$ (1 mM) in CH_3CN (a) and CH_3CN with 10% (vol.) NMP (b) during a CV (-0.5 to -2.3 V, 5 mV s^{-1}, CH_3CN (a) and -0.3 V to -1.7 V, 2.5 mV s^{-1} 10% (vol.) NMP (b)) with 0.1 M $TBAPF_6$ at a Au electrode under Ar recorded with ppp polarisation. The in situ measurement begins at the top of the figure, and ends at the bottom.

Electrochemical Measurements

Preliminary EIS measurements in the range 100 kHz – 1 Hz were conducted, and a frequency in the low region (10 Hz) was chosen to reflect the double layer capacitance and avoid convolutions due to electrolyte resistance at low electrolyte concentration[11,12] (0.1 mM $TBAPF_6$). Potentiostatic EIS was then measured at 10 Hz, starting from +1 V to -2 V vs Ag in 20 mV steps, with a 10 mV amplitude and 10 s equilibration time. The double layer capacitance can be approximated as a capacitance in series to the solution resistance, and the differential capacitance can be calculated from $C = -1/(2\Pi f Z_{Im})$, where C is the differential capacitance, f is the frequency, and Z_{Im} is the imaginary component of the measured impedance.[13]

Supplementary Note 2: Assignment of Spectral Bands

Table S1. Assignment of bands observed in the *in situ* VSFG spectra.

Peak positions as shown in Figure 3, have been obtained through fitting with Voigt functions; overlapping bands have been fitted using through a simultaneous combination of Voigt functions (see Figure S3), whereas isolated bands were fit using a single Voigt function in a separate fitting step. As shown in Figures 2 and S1, the VSFG spectra obtained at the least negative potentials are remarkably similar in both solvents, and similar to those reported in our previous work.[7] VSFG spectra obtained at

-0.55 V are shown in Figure S2a and b in CH_3CN and 10%(vol) NMP, respectively. The main band at ~1900 cm^{-1} in CH_3CN is clearly asymmetric, and well fit through a combination of two Voigt functions. The higher wavenumber band shows strong potential dependence (tuning rate ~ 28 cm^{-1} V^{-1}) whereas the most intense, lower wavenumber band shows a far smaller potential dependence (Figure 2c). The wavenumber and tuning rate of the higher wavenumber band is consistent with CO adsorbed at bridged sites on the Au electrode,[14] denoted CO@Au, with the CO produced from reductive dissociation of Mo(bpy)CO_4 on the Au surface from CVs recorded prior to spectroelectrochemical measurements to ensure successful construction of the SEC cell. There is little evidence of the CO@Au band in NMP, (Figure S2b) despite CVs also being recorded prior to SEC measurements. A clear shoulder to the 1890 cm^{-1} band is observed at ~1868 cm^{-1} when NMP is present (Figure 2b), and another band is observed at ~1825 cm^{-1} in both solvents. *ν*(CO) vibrations of Mo(bpy)CO_4 in CH_3CN are observed at 2115, 1904, 1875 and 1832 cm^{-1}, Figure S1; the highest wavenumber mode is outside of the spectral window probed in this work, where as the remaining vibrations are in excellent agreement with the bands observed at ~1890, 1868 (NMP only) and 1825 cm^{-1} at -0.55 V, hence we assign all of these bands to *ν*(CO) vibrations of Mo(bpy)CO_4.

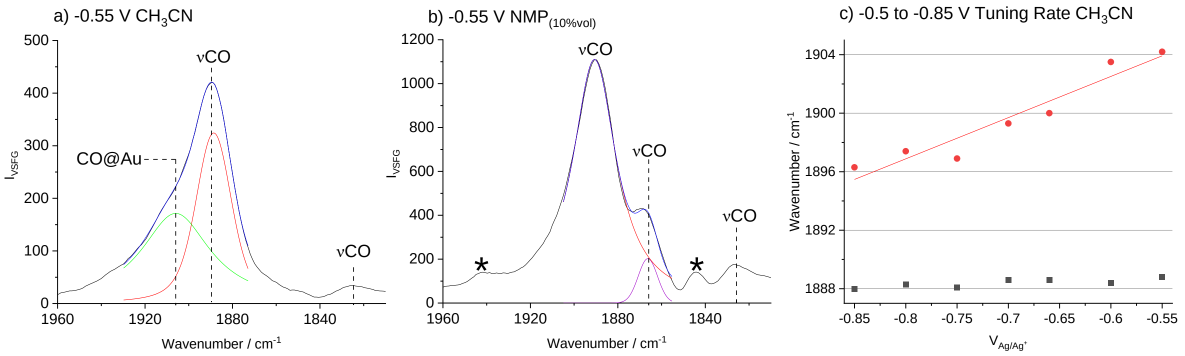

Figure S3. *Fits of the VSFG spectra obtained at -0.55 V in a)* CH_3CN *and b) 10%(vol) NMP. The broad baseline is a result of incomplete suppression of the non-resonant signal. Bands marked by an asterisk (*)are "phantom transitions," which arise as a result of absorption of mIR radiation by atmospheric water vapour through incomplete purging of the mIR beam path. c) Tuning rate of the overlapped vibrations at ~1900* cm^{-1} *in* CH_3CN *between -0.5 and -0.85 V; CO@Au, red circles and ν(CO) of Mo(bpy)*CO_4*, black squares.*

As the potential is swept more negatively, the intensity of the 1890 cm^{-1} *ν*(CO) of Mo(bpy)CO_4 continues to increase until -0.80 V in CH_3CN and -0.65 V in 10%(vol) NMP as Mo(bpy)CO_4 accumulates at the electrode surface. Between -0.6 V and -1.0 V, the most dominant band in 10%(vol) NMP switches, with the intensity of the ~1868 cm^{-1} band increasing simultaneously as the intensity of the 1890 cm^{-1} band decreases, Figures 1 and S2. Notably, the vibrational wavenumber of the ~1890 cm^{-1} *ν*(CO) band decreases over this potential range (Figure 3), indicating a change in local environment is occurring. Through close inspection of the VSFG spectra in CH_3CN, a weak band begins to form at

~1866 cm^{-1} (Figure 2b, Figure S4) between -0.8 and -1.0 V as the intensity of the ~1890 cm^{-1} ν(CO) of Mo(bpy)CO_4 band decreases; again this coincides with a shift to lower wavenumber of the latter vibration (Figure 3). Along with these changes, the ν(CO) band at ~1825 cm^{-1} disappears. Importantly in CH_3CN the 1890 cm^{-1} is dominant across the potential range of -0.55 to -1.0 V, whilst when NMP is present the dominant band switches from 1890 cm^{-1} to 1868 cm^{-1}, Figure S4.

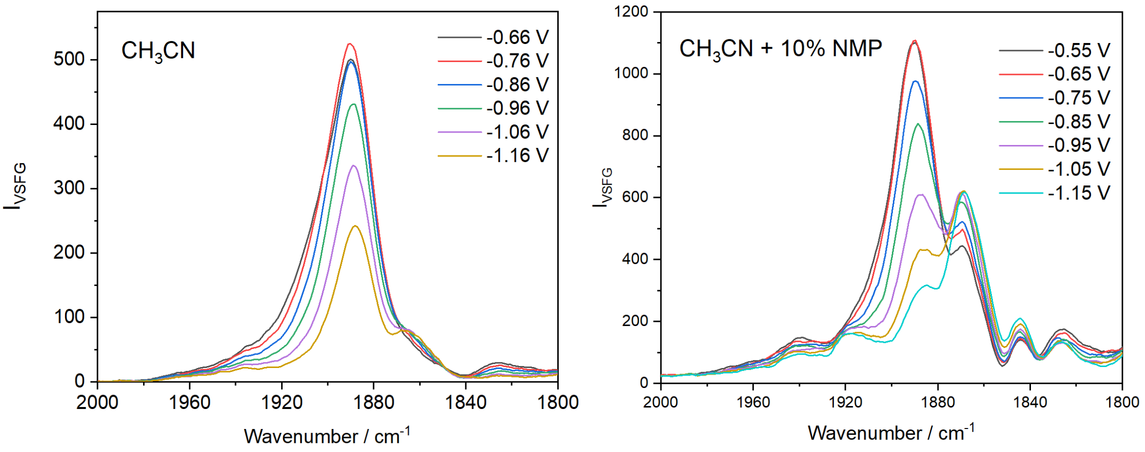

Figure S4. In-situ VSFG spectra obtained at the potentials indicated prior to R_1 for CH_3CN (left) and CH_3CN with 10% NMP (right).

As discussed in the main text, the change in the SEC behaviour does not coincide with a Faradaic process but does coincide with a change in capacitance. Hence we assign all vibrations between -0.5 and -1.0 V to ν(CO) of Mo(bpy)CO_4. Intensities of vibrational modes in VSFG spectroscopy are sensitive to the change in dipole moment and polarizability which occur during the vibration. On metallic surfaces, an additional selection rule is imposed: only vibrational modes whose change in dipole moment and polarizability that are perpendicular to the metal surface are VSFG active. Hence such changes in intensity of the ν(CO) modes of Mo(bpy)$(CO)_4$ is consistent with a reorientation of the double layer structure involving Mo(bpy)CO_4 species at, or close to, the electrode surface where the centrosymmetry of the bulk electrolyte is broken. Our *in situ* VSFG measurements suggest that this net reorientation indicated by the VSFG ν(CO) intensities, occurs in both CH_3CN and 10%(vol) NMP, but is clearly greater for the latter. From our experiments it is not possible to conclude whether the net reorientation in CH_3CN is smaller as a result of there being (i) a smaller population of Mo(bpy)$(CO)_4$ at, or close to, the electrode surface reorientating, or (ii) the difference in net reorientation between -0.55 and -1.0 V for each individual Mo(bpy)$(CO)_4$ molecule being lower.

In the vicinity of R_1 (-1.3 V) in CH_3CN the vibrational wavenumbers of the observed vibrations changed in intensity significantly; the ~1890 cm^{-1} band decreases a band at ~1860 cm^{-1} grows, Figure 3. In FTIR SEC measurements in THF, which employ a specialist SEC cell in which the signal is dominated by species in the bulk, away from the perturbing effects of the electrode surface, ν(CO) modes of $[Mo(bpy)CO_4]^{\bullet-}$ are reported at 1991 (outside of the spectral window of the current work) 1871, 1843 and 1805 cm^{-1}. The 1871 band of the complex in the bulk is in agreement with that observed following R_1 at ~1860 cm^{-1} at the electrode for the complex at the electrode in CH_3CN (Table S2).

At -1.6 V we see a weak band at ~1842 cm^{-1} appear in CH_3CN. When NMP is present, in the vicinity of R_1, a band at ~1845 cm^{-1} appears as the ~1868 cm^{-1} band (which is observed prior to R_1) decreases in intensity (Figure 1 and S2). By -1.6 V the band at 1845 cm^{-1} is the most intense feature. The potential at which the ~1845/1842 cm^{-1} band appears coincides with the onset of electrocatalysis (in the presence of CO_2) at -1.25 V in 10%(vol) NMP and -1.6 V in CH_3CN. The active electrocatalyst has been shown to be $Mo(bpy)CO_3^{2}$,[10] which has characteristic ν(CO) wavenumbers of 1846, 1725 and 1706 cm^{-1};[10] the highest wavenumber mode is in excellent agreement with the band observed at 1842 cm^{-1} and 1845 cm^{-1} in CH_3CN and 10%(vol) NMP, respectively (Table S1), unfortunately, the 1725 and 1706 cm^{-1} bands are obscured by solvent absorption (Figure S1). Owing to (i) the appearance of the ~1841/1845 cm^{-1} band in both solvents at the onset of electrocatalysis, (ii) the agreement in wavenumber to a ν(CO) mode of $Mo(bpy)CO_3^{2-}$ and (iii) similar potential independent behaviour of this band in both solvents, we assign the 1842/1845 cm^{-1} band to the same chromophore in both solvents: the active electrocatalyst $Mo(bpy)CO_3^{2-}$, despite the unfortunate coincidence in vibrational wavenumber of a ν(CO) mode of $Mo(bpy)CO_4^{\bullet-}$, noted above.

Table S1 indicates that the band at ~1860 cm^{-1} negative of R1 may either be due to (i) a concentration of $Mo(bpy)(CO)_4$ or (ii) $Mo(bpy)(CO)_4^{\cdot-}$. Both complexes have CO stretching modes at approximately the same frequency and it is not possible to distinguish the two scenarios. It is feasible that (i) $Mo(bpy)(CO)_4$ is present at potentials negative of R1 as we note the reduction potential is poorly defined in this solvent and fresh complex will be diffusing towards the electrode. Supporting the possible formation of $Mo(bpy)(CO)_4^{\cdot-}$ (scenario ii) which we propose then undergoes CO loss to form $Mo(bpy)(CO)_3^{2-}$ is the abrupt change in potential dependent CO frequency in the vicinity of R_1 (figure 3).

Table S1. *Reported ν(CO) vibrational wavenumbers of $[Mo(bpy)CO_4]$,* $[Mo(bpy)CO_4]^{\bullet-}$ *and* *$[Mo(bpy)CO_3]^{2-}$ complexes in THF.*[10]

	ν(CO), cm^{-1}			
$Mo(bpy)(CO)_4$	2012	1900	1882	1840
$[Mo(bpy)CO_4]^{\bullet-}$	1991	1871	1843	1805
$[Mo(bpy)CO_3]^{2-}$	1846	1725	1706	

Supplementary References

[1]. Clark, M. L.; Ge, A.; Videla, P. E.; Rudshteyn, B.; Miller, C. J.; Song, J.; Batista, V. S.; Lian, T.; Kubiak, C. P.; CO_2 Reduction Catalysts on Gold Electrode Surfaces Influenced by Large Electric Fields, *J.Am.Chem.Soc*., **2018**, 140, 17643−1765. DOI: 10.1021/jacs.8b09852

[2]. Neri, G; Donaldson, P. M.; Cowan, A. J.; *In situ* study of the low overpotential "dimer pathway" for electrocatalytic carbon dioxide reduction by manganese carbonyl complexes, ***Phys. Chem. Chem. Phys.***, 2019, **21**, 7389-7397. DOI: 10.1039/C9CP00504H

[3]. Neri, G.; Teobaldi, G.; Walsh, J. J.; Donaldson, P. M.; & Cowan, A. J.; *Detection of catalytic intermediates at an electrode surface during carbon dioxide reduction by an earth-abundant catalyst, Nature Catalysis*, **2018**, 1 (12), 952-959. DOI: 10.1038/s41929-018-0169-3

[4]. Ge, A.; Rudshteyn, Videla, P. E.; Miller, C. J.; Kubiak, C. P.; Batista, V. S.; Lian, T.; Heterogenized Molecular Catalysts: Vibrational Sum-Frequency Spectroscopic, Electrochemical, and Theoretical Investigation, *Acc. Chem. Res.* **2019**, 52, 1289–130 DOI:10.1021/acs.accounts.9b00001

[5]. Bhattacharyya, D.; Videla, P. E.; Cattaneo, M.; Batista, V. S.; Lian, T.; Kubiak, C. P.; Vibrational Stark shift spectroscopy of catalysts under the influence of electric fields at electrode– solution interfaces, *Chem. Sci.,* **2021**, 12, 10131. DOI: 10.1039/D1SC01876K

[6]. Gardner, A.M.; Saeed, K.; Cowan, A.J.; Vibrational Sum-Frequency Generation Spectroscopy of Electrode Surfaces: Studying the Mechanisms of Sustainable Fuel Generation and Utilisation. *Phys. Chem. Chem. Phys.,* **2019**, 21, 12067-12086. DOI: 10.1039/C9CP02225B

[7] Neri, G; Donaldson, P. M; Cowan, A. J., The Role of Electrode–Catalyst Interactions in Enabling Efficient CO_2 Reduction with $Mo(bpy)(CO)_4$ As Revealed by Vibrational Sum-Frequency Generation Spectroscopy, *J. Am. Chem. Soc.,* **2017**, 139, 13791−1379

[8]Lagutchev, A.; Lozano, A.; Mukherjee, P.; Hambir, S. A.; Dlott,D. D.; Compact broadband vibrational sum-frequency generation spectrometer with nonresonant suppression, *Spectrochim. Acta, Part A* **2010**,75, 1289−1296.

9. Rey, N.G.; Dlott, D. D.; Studies of electrochemical interfaces by broadband sum frequency generation, *J. Electroanal. Chem.*, **2017**, 800, 114–125. DOI:10.1016/j.jelechem.2016.12.023

[10] Tory, J.; Setterfield-Price, B; Dryfe, R. A. W; Hartl, F, [M(CO)4(2,2'-bipyridine)] (M=Cr, Mo, W) Complexes as Efficient Catalysts for Electrochemical Reduction of CO2 at a Gold Electrode, *ChemElectroChem*, **2015**, 2, 213 – 217. DOI: 10.1002/celc.201402282

[11] Mei, B.-A.; Munteshari, O.; Lau, J.; Dunn, B.; Pilon, L.; Physical Interpretations of Nyquist Plots for EDLC Electrodes and Devices, *J. Phys. Chem. C,* **2018**, 122, 194–206

[12] Khademi, M.; Barz, D. P. J.; Structure of the Electrical Double Layer Revisited: Electrode Capacitance in Aqueous Solutions, *Langmuir* 2020, 36, 16, 4250–4260.

[13] Lockett, V.; Sedev, R.; Ralston, J.; Horne, M.; Rodopoulos, T.; Differential Capacitance of the Electrical Double Layer in Imidazolium-Based Ionic Liquids: Influence of Potential, Cation Size, and Temperature, . *Phys. Chem. C* **2008**, 112, 7486–7495

[14] Chen, D.-J.; Allison, T. C., Tong Y. J.; Mechanistic Insights into Electro-Oxidation of Solution CO on the Polycrystalline Gold Surface as Seen by *in Situ* IR Spectroscopy, *J. Phys. Chem. C* **2016**, 120, 16132–16139
